# Eating for emotion regulation: Associations with threats to psychological needs from adolescents' peer, family, and academic daily stress

**DOI:** 10.1111/jora.70214

**Published:** 2026-06-04

**Authors:** Melanie J. Zimmer‐Gembeck

**Affiliations:** ^1^ School of Applied Psychology & Griffith Centre for Mental Health Griffith University Gold Coast Queensland Australia

**Keywords:** daily, emotion regulation, emotional eating, negative emotionality, stress

## Abstract

Food consumption can be an attempt to regulate negative emotion, positioning eating as a strategy for emotion regulation (*EER*). Given adolescents' developmental changes, such as sensitivity to stress and still maturing emotion regulation skills, adolescents may often rely on an accessible emotion regulation strategy, such as EER, when they experience stress. However, there are few studies of stress, emotion, and emotional eating in adolescents, and no studies have directly measured EER. The primary aim of this study was to determine whether adolescents' daily reports of EER were associated with academic, peer, and family stressors. Australian adolescents (*N* = 319, *M*_age_ = 13.9 years, 45% boys, 65% White, 85% born in Australia) completed a 7‐day daily survey via an app to report their EER, the biggest problem of the day (*stressor*), and stressor intensity and domain. They also reported how much stressors threatened their psychological needs for relatedness, competence, and autonomy (*stressor threat*) as described in self‐determination theory. Dynamic structural equation modeling (DSEM) was used to account for the multilevel data, while estimating within and across‐day associations between (1) stressor intensity and EER in one model and (2) stressor threat and EER in a second model. DSEM results showed that, after controlling for within‐day associations and stress chronicity, adolescents who rated their stress as more intense and threatening reported elevated EER the next day and, conversely, a greater degree of EER related to higher ratings of stressor intensity and threat the next day. In addition, reports of EER were elevated on days when adolescents reported peer or family stress compared to days without, with no difference for academic stress. Overall, EER and stressor intensity and threat are intertwined and bidirectionally influential, and interpersonal stress may be a particular risk for EER. Future research could determine whether EER foretells later disordered eating and declining well‐being.

## INTRODUCTION

The rising rate of obesity among children, adolescents, and adults, alongside the possibility of an interface of emotions with eating, has prompted decades of research on emotional eating. Emotional eating has been described as food consumption, often highly palatable snack foods, that occurs when emotions are elevated (Hsu & Raposa, 2021). A major focus of this research on emotional eating has been on the role of eating as a strategy to soothe and regulate negative emotion, particularly in relation to stress. This approach positions emotional eating as a possible strategy for emotion regulation, with emotion regulation broadly defined as the physiological, cognitive, or social actions individuals use to recognize, manage, and modify emotional experiences (Cole et al., 2004; Nolen‐Hoeksema, 2012). With development, emotion regulation coheres into a suite of complex actions especially necessary for adaptation when facing negative emotions associated with stressful events (Skinner & Zimmer‐Gembeck, 2016, 2023).

Although much of the existing research has involved university students or adults, early and middle adolescence (age 10–16) are possibly the most important developmental periods for investigating stress in relation to emotional eating. Adolescents are especially reactive to stress for many physiological and social‐environmental reasons, such as hormonal and neural changes, social status pressures, and increasing opportunities for autonomy (Filetti et al., 2024; Romeo, 2014). These maturational conditions inflame adolescents' attention to social rewards, leaving them vulnerable to intense negative emotions in reaction to social and status‐relevant threats from stressful experiences like peer rejection and victimization (Crone & Dahl, 2012; Lee et al., 2020; Silk et al., 2014; Somerville, 2013; Zimmer‐Gembeck, 2016). Such hypersensitivity is tied to adolescents' elevated risk for the onset of internalizing mental health problems.

At the same time that stress sensitivity is heightened, adolescents' emotion understanding and emotion regulation skills are still maturing (Blakemore & Mills, 2014; Fombouchet et al., 2023; Skinner & Zimmer‐Gembeck, 2016, 2023). As a result, emotional reactions to stressors can be intense, while emotion regulation skills remain rather rudimentary. Under such circumstances, adolescents may be especially likely to rely on readily available, less active strategies and turn to rewards that quickly soothe negative emotions. Eating palatable foods may serve this function well. Furthermore, adolescents often want to be more self‐reliant when coping with stressful events when compared to children, which can introduce additional difficulties with effective emotion regulation and increase avoidance as a coping response (Young & Limbers, 2017; Zimmer‐Gembeck & Skinner, 2011). The increased desire and ability for self‐reliance extends to eating, with adolescents having more opportunity than children to select what, when, and how much to eat (Bassett et al., 2008). Together, such developmental and social circumstances create conditions that may encourage emotional eating in response to stressors among adolescents, and these converging influences help explain why emotional eating often onsets and becomes prevalent during adolescence (Jääskeläinen et al., 2014; Joseph et al., 2023). Moreover, greater emotional eating in adolescence may signal broader difficulties with emotion regulation and may represent an early and important risk factor for later disordered eating and associated difficulties with physical health and emotional adjustment.

Emotional eating may also exacerbate adolescents' sensitivity to future stress. Adolescence is marked by physical changes associated with puberty, and concerns and anxiety about appearance (including body shape and size) and body dissatisfaction (Stice, 2003; Zimmer‐Gembeck et al., 2018). Concerns and dissatisfaction arise from physical changes but are also related to adolescents' emerging understanding of the importance of their physical appearance to others and the fact that attractiveness, displayed offline and online, often brings higher social status in adolescents' peer groups and beyond (Nesi & Prinstein, 2019; Trekels et al., 2024). Consequently, adolescents' emotional eating may not only be a response to stress but may also instigate appearance‐related concerns that increase sensitivity to stressful events into the future. The aim of the present study was to examine bidirectional associations between stressful events and emotional eating in the daily lives of adolescents, with a specific focus on eating as a strategy to regulate negative emotion. This behavior is referred to as eating for emotion regulation (*EER*).

### Emotional eating and eating for emotion regulation: definitions and links to stress

The evidence linking emotion to eating is far from consistent across studies (Bongers & Jansen, 2016). In addition, most of these findings have been derived from research with university students and adults. Such research suggests that only *some* individuals consume more food when they feel more emotional (including feeling more positive and negative emotion; e.g., for a review, see Evers et al., 2018), and studies often report very little association between emotion and observed or reported eating behavior, even among self‐described emotional eaters (Ahlich et al., 2023). Yet, *perceived* emotional eating continues to raise concerns, with associated attempts at intervention, because it can result in increasing negative affect (Hsu & Raposa, 2021; Stammers et al., 2020). Other concerns include using food to regulate negative emotion in place of more effective strategies to cope with or regulate distress (Stice et al., 2002), and associated self‐deprecating thoughts, shame, and guilt (Macht & Dettmer, 2006; Popless‐Vawter et al., 1998; Wansink et al., 2003; Wong & Qian, 2016).

One explanation for weak and inconsistent findings regarding emotion and eating may be the lack of clarity in the definition of emotional eating. For example, some definitions of emotional eating refer to only negative emotions and their links to eating, whereas others consider both negative and positive emotions (Ahlich et al., 2023; Bongers & Jansen, 2016; Cardi et al., 2015). Some definitions mention overeating (Evers et al., 2018; Romeijn et al., 2021), but others refer to a general increased desire to eat, eating behavior in general, or a change in eating (Barnhart et al., 2024; Chawner & Filippetti, 2024; Fuente González et al., 2022; Macht, 2008). Some definitions identify eating as a strategy for emotion regulation as an important feature (Heatherton & Baumeister, 1991; Macht, 2008), but others make no mention of emotion regulation. Finally, in some work on emotional eating, stressful events are expected to be the impetus for the negative mood states linked to food consumption, with emotional eating sometimes called “stress eating” or stress‐induced eating (Reichenberger et al., 2018, 2021; Stammers et al., 2020). For example, escape theory applied to emotional eating conceptualizes eating as a means of escaping or diverting attention from aversive stressors or threats perceived as uncontrollable or excessively threatening to self‐beliefs or social relationships (see Spoor et al., 2007).

Given the various definitions of emotional eating, there have been many approaches to measuring it (Ahlich et al., 2023). However, at their core, most existing survey measures ask about a desire to eat in response to emotions or ask for retrospective reports of eating when feeling certain emotions (e.g., sadness, loneliness, boredom). Notably, in other research, emotional eaters have been described as using food *as an emotion regulation* strategy (Macht, 2008). It may be this —often unmeasured—aspect of using eating as a strategy for emotion regulation, or EER, that is most linked to negative emotion, and to future disordered or problem eating behaviors and later physical and emotional health problems. In this study, EER was conceptualized as a strategy to influence the intensity, experience or expression of negative emotions (see Gross, 1998, p. 275).

Affect regulation models have also been applied to understand stress and eating, proposing that individuals who experience stronger emotional reactivity to stressors are more likely to perceive themselves as engaging in emotional eating‐‐that is, depending more on food to alleviate or soothe negative affect (Hawkins & Clement, 1984; Heatherton & Baumeister, 1991). This pattern often involves the consumption of high‐fat/sugary/energy‐dense foods to avoid, reduce, or comfort negative affect following stressful events. In addition, difficulties in emotion regulation have been identified as a potential mechanism underlying (or closely associated with) emotional eating (Chawner & Filippetti, 2024; Crockett et al., 2015; Evers et al., 2010; Gouveia et al., 2019; Shriver et al., 2019), and greater difficulties in emotion regulation are reliably linked with more disordered eating patterns (Zhou et al., 2025). Measuring EER, particularly during adolescence, may yield novel insights into how stressful experiences become linked with eating behavior and identify modifiable risks that could be addressed to reduce future eating and emotional problems.

### Stress, emotional eating, and EER in adolescents

When events exceed coping resources, they are described as stressors because they yield negative emotion and attempts to change the provoking situation or modify the negative emotional response (Lazarus, 2006; Lazarus & Folkman, 1984). Although few studies of adolescents could be located, research has been conducted with the aim of identifying whether stress is a risk for elevated eating or emotional eating (Debeuf et al., 2018; Hsu & Raposa, 2021). In three cross‐sectional studies of adolescents from the USA, Europe and Australia, adolescents' perceived level of stress was associated with greater self‐reported emotional eating, measured as a greater desire to eat when emotional or a report of eating more when feeling negative emotions (Vandewalle et al., 2014; Webb et al., 2021; Young & Limbers, 2017). Three other studies focused on adolescent stress in the form of parent or maternal rejection in experimental and diary study designs (Vandewalle, Mabbe, et al., 2017; Vandewalle, Moens, et al., 2017) or appearance teasing by parents (Webb et al., 2021) in a longitudinal study, finding that these interpersonal stressors are related to elevated levels of adolescents' emotional eating.

One notable enhancement to research in this area has been the use of experience sampling methods (ESM; Bongers & Jansen, 2016). These methods are useful for understanding dynamic associations of events, like stressors, that can fluctuate over hours or days (Shiffman et al., 2008). To date, there have been a few ESM studies focused on adolescents' stress and eating, but in those that have been conducted, they have supported links between stress, emotion, and reports of eating‐related thoughts or behaviors among adolescents. In addition to Vandewalle, Mabbe, et al. (2017) mentioned above, three other ESM studies were located. Two studies conducted in the USA examined daily stress and emotional experience as correlates of adolescents' reports of their eating behavior (Heshmati et al., 2022; Hsu & Raposa, 2021), and the other study examined stress and desire to eat or snacking behavior among Belgian adolescents (Debeuf et al., 2018). Although not always finding daily associations, all studies found evidence of the impact of negative life events or feeling “stressed” on reports of greater food intake or a greater desire to eat. None of these studies asked about EER directly or about the details of stressful events, such as the level of threat or the type (domain) of stress.

### Domains of stress for adolescents and threat appraisals

The domain of stress, such as work or interpersonal stress, has been found to be an important consideration in studies of stress and eating among adults (for a review, see O'Connor & Conner, 2011). In this research, ego‐threatening stressors, which involve a fear of failure and threats to feelings of self‐competence or self‐esteem such as stress from work, has been more strongly associated with eating behavior when compared to the impact of physical stress (Hetherington et al., 1992). However, other research identifies the even stronger impact of interpersonal stress on eating behavior when compared to ego‐threatening stress in adults (Tanofsky‐Kraff et al., 2000). Relations of stress with emotional eating or eating behavior by domain of stress have not been investigated in adolescents. Instead, adolescent research has concentrated on one specific domain of stress (e.g., family stress) or on “stressors” without mentioning domain. In the current study, we considered stress across three domains that are important to most adolescents, including academics, family, and peer stressors. Academic stressors were expected to align best with ego‐threatening stressors, whereas family and peer problems were considered interpersonal stressors.

Adolescents' appraisals of stressor intensity and threat across and within domains of stress were of interest as instigators and outcomes of daily EER. Intensity of stress was a rating of how “bad” the stressor was for the adolescent. Threat was an adolescent's appraisal of how much the stressor undermined feelings of relatedness, competence, and autonomy. The consideration of threat was founded on self‐determination theory, which identifies how experiences that meet the human psychological needs of relatedness, competence, and autonomy are the underpinnings of human motivation, adaptive or maladaptive behavior, and well‐being (SDT; Deci & Ryan, 1985; Ryan & Deci, 2017). When experiences do not meet these psychological needs, they increase negative emotion and spark regulation and coping responses consistent with the definition of stress (Skinner & Wellborn, 1994; Zimmer‐Gembeck et al., 2009; Zimmer‐Gembeck & Skinner, 2024). Thus, in this study, the intensity and threat to psychological needs for relatedness, competence, and autonomy were measured as indicators of the significance of daily stressors for adolescents.

### The current study and research hypotheses

Although findings are mixed and criticisms are emerging regarding the conceptualization of emotional eating, stress continues to be of interest as a possible correlate of emotional eating in adolescents and adults. However, no studies have focused on EER, which may be a specific negative indicator of problems with stress responding. Moreover, this association is important to study among adolescents, a developmental stage when adaptive responses to stress are still under development, and problems with emotion regulation may onset and become more resistant to change in the future. Thus, EER may be an indicator of future problems with disordered eating and emotional adjustment. Also relevant to this study, it is not known whether stressor intensity and threat in the domains most salient to adolescents—peer relationships, family, and academics—play a role in EER. The current study investigated the interrelationships of adolescents' EER with experience of their “worst” daily problems (i.e., stressors). This included examining reports of stress domain, stressor intensity, and how adolescents appraised the threat level of the stressors. Using dynamic structural equation modeling (DSEM; Asparouhov et al., 2018) to account for the multilevel data (nesting of assessment days within persons), dynamic associations between stress and EER within each day and from day to day were simultaneously estimated, while also controlling for the chronicity of stress. The following hypotheses were tested:

1. Stressor intensity and EER reported on the same day will be positively correlated.
2. Controlling for the association between stressor intensity and EER on the same day and stress chronicity, there will be a positive association between stressor intensity on one day and EER on the next day, and (conversely) a simultaneous positive association between EER on one day and stressor intensity on the next day.
3. Stressor threat to psychological needs and EER reported on the same day will be positively correlated.
4. Controlling for the association between stressor threat and EER on the same day and stress chronicity, there will be a positive association between stressor threat on one day and EER on the next day, and (conversely) a simultaneous positive association between EER on one day and stressor threat on the next day.
5. Greater stressor intensity and threat in each domain (peer relationships, family, and academic) will be associated with a greater degree of EER. However, stress intensity and threat in the interpersonal (peer and family) domains will have stronger relations with EER than stress intensity and threat in the academic domain.

## METHOD

### Participants

Participants (*N* = 319; *M*_*age*_ = 13.91, *SD*_age_ = 1.52; 45% boys) were randomly selected for this study from a larger pool of 863 participants recruited for a longitudinal study. Baseline (T1) data had been collected from students of three Australian secondary schools (grades 7 to 10) and five of their feeder primary schools (grades 5 and 6). Across the schools, 52% of students had returned consent forms and, of these, 80% of parents gave informed consent. All participants with parent consent gave assent before participation. Most parents of adolescents in the longitudinal study (*n* = 677; 78%) agreed to be contacted for future research. To be representative of the full range of emotional problems reported, two strata were formed based on adolescents' high or low scores on depressive and social anxiety measures, and adolescents were then randomly selected from these strata. Parents and adolescents (326 of 465 contacted, 70%) who gave consent were introduced to the study protocol and chose a Monday to start the 7‐day daily diary. Seven participants were excluded from this study because they only participated on the first day, leaving a sample size of 319. The aim was to include 300 participants to have the power to detect moderate associations across days.

Adolescents could select multiple of the following to describe themselves: White; Asian; Australian First Peoples, Torres Strait Islander or Pacific Islander; other race/ethnicity; born in Australia; and born in New Zealand. Overall, 99% of adolescents ticked at least one answer, with 65% reporting White, 9% Asian, 5% Australian First Peoples, Torres Strait Islander or Pacific Islander, 37% other race/ethnicity, 85% born in Australia, and 7% born in New Zealand. In their statistics, schools reported that the proportion of the student population who spoke a language other than English at home ranged from 5%–29%, and 14%–29% of students were in the lowest, and 4%–30% in the highest, income quartiles.

### Procedure

Prior to data collection, approval was obtained from Griffith University Human Research Ethics Committee. All participants had their own smartphone or tablet for use and were guided through installing an app on their device to capture their daily responses. Prior to the start of the first day, participants received a text reminder and then daily reminders about survey completion were sent at 6:00 pm (when the survey link opened) and 7:30 pm each evening. If the survey remained incomplete, another reminder was sent at 8:30 pm. The opportunity for completing the daily survey closed at 11 pm. After completion of the 7 days, each participant received a gift voucher ($5/day plus an extra $5 for completing all 7 days). For each completed day, participants were entered into 1 of 3 prize draws for another $100 gift voucher. Data recruitment and collection started in October 2021 with most data collected from March–May 2022, with a small group delayed in their participation until early August 2022. Data collection was suspended during the exam period of late November/early December, summer break (December–January), and the first month of school (February). This study was not preregistered. This report includes a description of how sample size was determined, all data exclusions (if any), all manipulations, and all measures in the study. The data and code are available upon request from the corresponding author.

### Measures

#### Eating for emotion regulation (EER)

Each day, adolescents reported how much they ate to feel better using one item, “How much did you eat today to try to make yourself feel better or to help with your emotions?” Responses could range from 1 (*Not at all*) to 7 (*Extremely*). The item was worded to capture behavior implied in the definition of emotion regulation: Emotion regulation is “the process by which individuals influence which emotions they have, when they have them, and how they experience and express these emotions” (Gross, 1998, p. 275). The response options were selected to align with responses in measures of emotion regulation strategies or ways of coping with stress that ask about amount of use, while also avoiding the possibility of low variability if adolescents were asked to report on their frequency of eating (none, once, etc.; or “used once”, “used frequently”, etc.). The item was also designed to avoid measuring the amount of food consumed (i.e., binge eating). The item was correlated with the 7‐day average scores of “worst” level of sadness, worry, and anger reported across the days, *r*s ranged from .19 to .24, all *p* < .001, but had weaker correlations with the 7‐day average scores of end of day reports of sadness, worry, and anger, *r*s ranged from .10 (*p* > .05) to .17 (*p* < .01). EER was also positively correlated with 7‐day average avoidance coping (*r* = .19, *p* < .001) and 7‐day average rumination (*r* = .29, *p* < .001).

#### Stressor intensity, domain, chronicity, and threat

Following an open‐ended question to describe the worst problem or stressor of their day (e.g., receiving marks for recently submitted assignment; Mum yelling at me; no one listening to anything I have to say; I feel like my friends don't want to be my friends anymore), adolescents rated the intensity of the stressor (*Overall, how bad was this problem?*) on a scale from 1 (*not at all bad*) to 7 (*extremely bad*). After this, three items asked adolescents to classify the problem as related to peer relationships (*Did this problem involve a friend or classmate?*; 0 = no, 1 = yes), family (*Did this problem involve a parent or sibling*; 0 = no, 1 = yes), and/or academics/school (*Did this problem involve school or schoolwork*?; 0 = no, 1 = yes). This was followed by a question to measure stressor chronicity (*Did the problem start today?*; 1 = yes, 2 = no, earlier this week, or 3 = no, more than a week ago). Finally, adolescents appraised how much the problem threatened their psychological needs for relatedness (*Did it make you feel rejected, excluded, or that it might damage your relationships?*), competence (*Did it make you feel dumb or stupid?*), and autonomy (*Did it make you feel pressured, bossed, pushed around, or limited in what you could do?*), using responses from 1 (*not at all*) to 7 (*extremely*), similar to what has been used in previous research (Zimmer‐Gembeck et al., 2009; Zimmer‐Gembeck & Skinner, 2024). A composite score was formed by averaging the three threat items within each day given (1) moderate correlations between the threats (*r* of .5 or higher), (2) a high Cronbach's *α* (of the three average threat scores across the 7 days) of .86, and (3) that stressors often threaten more than one need (e.g., being behind on an school assignment can be a threat to competence but also can threaten autonomy because it can feel coercive and limiting; a conflict with a peer or a parent can threaten both relatedness and autonomy).

### Overview of the data analyses

Prior to addressing the aims of the study, data were examined for possible data quality problems and 7 participants were excluded because they had stopped participating after the first day. In total, 2034 daily diary entries of 2223 possible (91%) from 319 adolescents were available, but 1717 daily entries of 1914 possible (90%) were used for analyses of associations across days, because the first day reports could not be included as outcome variables. To examine missingness, a count of the number of days each adolescent missed the survey was created and this was correlated with other measures. There were no significant associations of missingness with any of the 7‐day average of the main measures (EER, stress chronicity, stressor intensity, and stressor threat, *r* ranged from −.03 to .11, all *p* > .05). Missingness was not associated with gender, *r* = −.08, *p* = .163. However, missingness was associated with age, *r* = .22, *p* < .001. Thus, older adolescents missed more days of the survey.

**FIGURE 1:**
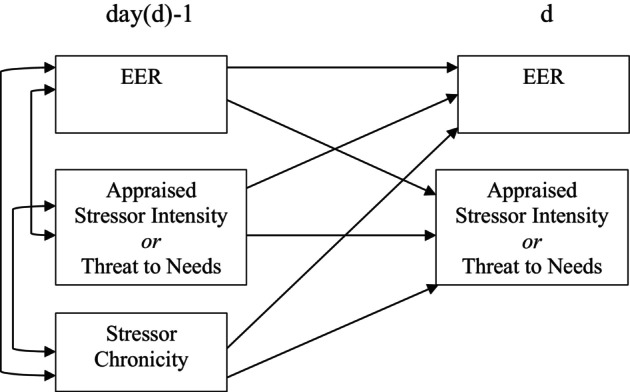
Illustration of the dynamic path model fit to estimate the within‐day association of eating for emotion regulation (EER) with stressor intensity or threat to needs and bidirectional across‐day associations of EER with stressor intensity or threat to needs.

Descriptive statistics and correlations between measures (averaged across all days) were estimated focusing on overall stress ratings and stress ratings within the peer, family, and academic domains as related to EER. Mplus v8.10 was used to fit multilevel dynamic structural equation models (DSEMs). Each DSEM estimated within‐day associations and day‐to‐day associations simultaneously (see Figure 1). Also, stressor chronicity was controlled for in all models. Thus, Model 1 tested (1) within‐day associations of EER with stressor intensity, (2) the association of EER reported on the previous day (time d‐1) with stressor intensity reported on the following day (time d), and (3) the converse association of stressor intensity reported on the previous day (time d‐1) with EER reported on the following day (time d). Model 2 was the same as Model 1, but stressor intensity was replaced with stressor threat.

Finally, EER on days where peer relationship stress was the biggest problem of the day was compared to days when it was not. These comparisons were repeated for family stress and for academic stress. The hypotheses for this study were specified before the data were collected. However, it was not clear whether comparisons and analyses of EER within stressor domains could be conducted until the level of stress in each domain could be determined.

## RESULTS

### Descriptive statistics and correlations across the week

Table 1 presents the means and standard deviations of measures. On average across the days, adolescents reported a moderate level of EER and low to moderate levels of stressor intensity and threat. Also, on average, stressors had started that day (i.e., they were not “chronic”) and 53% of participants reported *only* about stressors that started on the day of reporting.

**TABLE 1:** Correlations between all measures when averaged across the days with means (*M*) and standard deviations (*SD*) (*N* = 319).

		1	2	3	4	5	6	7	8	9	10
1	EER	–									
2	Stressor intensity	.27***	–								
3	Stress chronicity	.10	.15**	–							
4	Stressor threat	.25***	.65***	.21***	–						
5	Peer stressor intensity^a^	.06	.51***	.02	.41***	–					
6	Peer stressor threat^a^	.12*	.48***	.09	.69***	.77***	–				
7	Family stressor intensity^a^	.18**	.53***	.03	.38***	.43***	.38***	–			
8	Family stressor threat^a^	.16**	.43***	.16**	.67***	.37***	.54***	.75***	–		
9	Acad stressor intensity^a^	.17**	.63***	.11	.45***	.37***	.33***	.32***	.29***	–	
10	Acad stressor threat^a^	.22***	.55***	.17**	.77***	.38***	.57***	.32***	.49***	.72***	–
	*M*	2.56	3.35	1.02	2.23	2.62	1.78	2.52	1.66	3.34	2.20
	*SD*	1.27	1.16	0.64	1.05	2.24	1.69	2.26	1.68	1.69	1.33

^a^ A score of 0 was assigned to the participants who reported no problem within the domain across all days. Acad, Academic; ER, for emotion regulation.

* *p* < .05.

** *p* < .01.

*** *p* < .001.

Table 1 also summarizes correlations between measures, after each measure had been averaged across the 7 days for each participant. Providing preliminary support for H1–H4, stressor intensity and threat to needs were associated with a greater degree of EER. Also, stress was rated as more intense when it was more chronic and when adolescents perceived more threat. There were significant positive associations between EER, stressor intensity, and stressor threat when averaged across the days for each stressor domain (excepting a nonsignificant association of peer stressor intensity with EER), providing preliminary support for H5. In addition, girls reported higher stress intensity (*r* = .27, *p* < .001) and threat (*r* = .25, *p* < .001). Age had small negative associations with peer and family stressor intensity (*r* = −.12 and *r* = −.13, respectively, both *p* < .05).

### Domains of stress

Of all participants, 212 (66%) reported peer stress on at least one day, with 20% reporting it one time only. Forty‐five adolescents (14%) reported peer stress was their biggest problem every day. For family stress, 204 (64%) reported it on at least 1 day, with 20% reporting it one time only. No adolescents reported family stress was their biggest problem every day, but one reported it on 6 days. For academic stress, 286 (90%) reported school/academic stress, with 21% reporting it on 1 day only. Three (1%) reported academic stress every day, and 12 more (13%) reported it on 6 days. Overall, 14 participants (4%) never reported a stressor that involved peers, family, or academics. On any given day, between 24% (Monday) and 46% (Saturday) reported their biggest problem of the day did *not* involve peer, family, or academics.

When intensity and threat were averaged across the 7 days within peer, family or academic stress (assigning a score of 0 to participants who never reported a problem in a domain to maintain all 319 participants), the average level of academic stressor intensity (*M* = 3.34; see Table 1) was significantly higher compared to peer (*M* = 2.62), paired *t*(318) = −5.73, *p* < .001, and family (*M* = 2.52) stressful events, paired *t*(318) = −6.20, *p* < .001. Peer and family stressor intensity did not differ, paired *t*(318) = 0.71, *p* = .478. Also, the average level of academic stressor threat (*M* = 2.20) was significantly higher compared to peer (*M* = 1.78), paired *t*(318) = −5.28, *p* < .001, and family (*M* = 1.66), paired *t*(318) = −6.24, *p* < .001. Peer and family stressor intensity did not differ, paired *t*(318) = 1.27, *p* = .206.

### Associations of stressor intensity with EER within and across days

**FIGURE 2:**
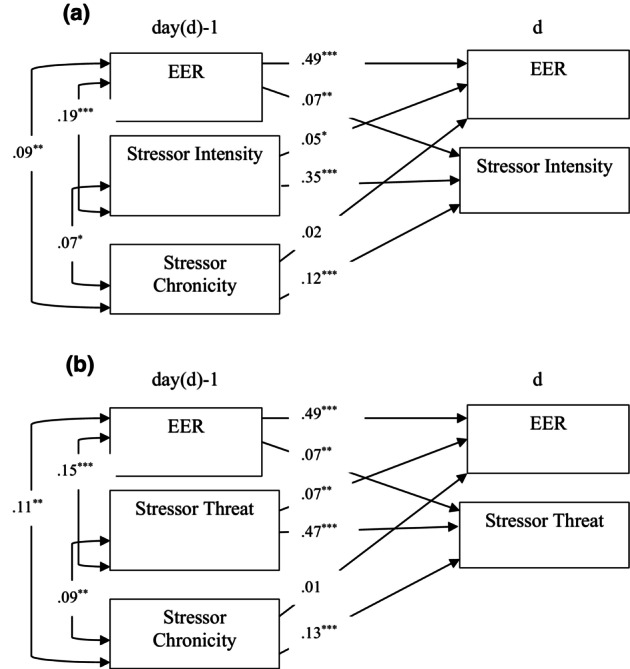
Results of the dynamic path models fit estimating within‐day association of eating for emotion regulation (EER) with stressor intensity (a) and threat to needs (b) and bidirectional across‐day associations of EER with stressor intensity (a) and threat to needs (b).

A first DSEM was fit to estimate within‐ and across‐day bidirectional associations between daily stressor intensity and EER, while controlling for stress chronicity (see Table 2, see Figure 2a). Supporting H1, stressor intensity (*β* = .19, *p* < .001) and stress rated more chronic (*β* = .09, *p* = .009) were associated with a greater degree of EER on the same day. In addition, stressor intensity was higher when chronicity was higher (*β* = .07, *p* = .047). Above and beyond these within‐day associations, there were also significant and positive bidirectional associations between stressor intensity and EER from 7 day to the next, which supported H2. Controlling for EER on the previous day, stressor intensity on the previous day had a small positive association with EER the next day (*β* = .05, *p* = .040). At the same time, controlling for stressor intensity on the previous day, EER on the previous day was significantly positively associated with greater stressor intensity the next day (*β* = .07, *p* = .002). Stress chronicity was also related to next‐day stressor intensity (*β* = .12, *p* < .001), but chronicity was not uniquely associated with next‐day EER (*β* = .02, *p* = .483). The model of these daily interrelations accounted for 25% of the variance in EER and 16% of the variance in stressor intensity. The model was just‐identified, so no fit statistics were available. However, after removing the nonsignificant path from stress chronicity to next‐day EER, model fit statistics were available and the model fit the data well, *χ* ^2^(1) = 0.49, *p* = .486, CFI = 1.00, TLI = 1.00, SRMR (within) = .003, SRMR (between) = <.001.

**TABLE 2:** Results of two dynamic models of bidirectional across day and within‐day associations of stress appraisals (Model 1 stressor intensity and Model 2 stressor threat to psychological needs) with eating for emotion regulation (EER), also considering stress chronicity (*N* = 319).

Paths	Model 1, all stressor types^a^		Model 2, all stressor types^a^	
	Stress appraisal = Stressor intensity		Stress appraisal = Stressor threat	
	*β*	*p*	*β*	*p*
Bidirectional paths (Day T − 1 → Day T)				
Stress appraisal → EER	.05	.041	.07	.006
EER → Stress appraisal	.07	.002	.07	.002
Stress chronicity → Stress appraisal	.12	<.001	.13	<.001
Stress chronicity → EER	.02	.483	.01	.591
Concurrent associations (within day)				
EER with Stress appraisal	.19	<.001	.15	<.001
EER with Stress Chronicity	.09	.009	.11	.009
Stress appraisal with Stress Chronicity	.07	.047	.09	.008
Autoregressive paths (Day T − 1 → Day T)				
EER	.49	<.001	.49	<.001
Stress appraisal	.35	<.001	.47	<.001

*Note*: Model 1: Stressor intensity *R* ^2^ = .16, EER *R* ^2^ = .25. Model 2: Stressor threat *R* ^2^ = .26, EER *R* ^2^ = .26.

^a^ All stressors: all types of worst problems of the day, with 66% of all reported stressors involving peer, family or academic stress.

### Associations of stressor threat with EER within and across days

A second DSEM was fit to estimate within‐ and across‐day bidirectional associations between stressor threat (composite of threat to relatedness, competence, and autonomy across all stressor domains) and EER, while also controlling for stress chronicity (see Table 2, see Figure 2b). Supporting H3, a higher threat rating (*β* = .15, *p* < .001) and (as in the model above) stress rated more chronic (*β* = .11, *p* = .009) were associated with a greater degree of EER on the same day. In addition, stressor threat was higher when chronicity with higher (*β* = .09, *p* = .008). Above and beyond these within‐day associations, there were significant and positive bidirectional associations between stressor threat and EER from 1 day to the next, providing support for H4. Controlling for EER on the previous day, stressor threat on the previous day was associated with a greater degree of EER the next day (*β* = .07, *p* = .006). At the same time, controlling for stressor intensity on the previous day, EER on the previous day was significantly associated with greater stressor threat the next day (*β* = .07, *p* = .002). Stress chronicity was also related to next‐day stressor threat (*β* = .13, *p* < .001), but chronicity was not uniquely associated with next‐day EER (*β* = .01, *p* = .591). This model accounted for 26% of the variance in EER and 26% of the variance in stressor threat. The model was just‐identified, so no fit statistics were available. However, after removing the nonsignificant path from stress chronicity to next‐day EER, model fit statistics were available and the model fit the data well, *χ* ^2^(1) = 0.34, *p* = .561, CFI = 1.00, TLI = 1.00, SRMR (within) = .003, SRMR (between) = <.001.

### Stress by domain: Peer, family, academic

EER was compared between stressor domains within person. Partially supporting H5, adolescents scored higher in EER on days when peer stress was their biggest problem (*M* = 2.53, *SD* = 1.34) than days when it was not (*M* = 1.75, *SD* = 1.77), paired *t*(318) = −7.72, *p* < .001. A similar difference was found on days when a family stressor was the biggest problem (*M* = 2.56, *SD* = 1.30) compared to days when it was not (*M* = 1.71, *SD* = 1.74), paired *t*(318) = −9.24, *p* < .001. The difference in EER was not significant for days when an academic stressor was reported as the biggest problem (*M* = 2.43, *SD* = 1.56) relative to days when it was not (*M* = 2.38, *SD* = 1.46), paired *t*(318) = 0.73, *p* = .465.

## DISCUSSION

Collecting data from adolescents each day for 7 days, the aim of the present study was to examine associations of eating for emotion regulation (*EER*) with adolescents' appraisals of intensity and threat from stressful events. The threats reflected the three core psychological needs of relatedness, competence, and autonomy identified within self‐determination theory (Deci & Ryan, 1985) and theoretical extensions of this theory into stress‐coping (Skinner & Wellborn, 1994; Skinner & Zimmer‐Gembeck, 2016). The analyses focused on the most intense (“biggest”) problem (stressor) of the day and also compared EER on days with and without subtypes of stressful events that fell within the domains of peer relationships, family, and academics.

### Between‐person and within‐person bidirectional associations

When between‐person correlations were examined, adolescents who reported a greater degree of EER across a week also appraised their biggest problems as more intense and more threatening to their psychological needs for relatedness, competence, and autonomy. In addition, EER and more negative appraisals of daily stressful events occurred in tandem within a day for adolescents, with EER elevated on days when a more intense and threatening stressful event was reported. Further, taking better advantage of the daily reports, dynamic modeling supported associations of higher stressor intensity and threat with a greater degree of EER on the following day. Also, revealing bidirectional associations, EER was associated with reports of greater stressor intensity and threat the following day.

Somewhat stronger effects were found for the impact of EER on stressor intensity the next day than the reverse, although the bidirectional links were similar in strength for stressor threat. These findings align with prior research demonstrating a link between general stress and emotional eating in both adolescents (Hsu & Raposa, 2021; Vandewalle, Mabbe, et al., 2017) and adults (Hill et al., 2018, 2022; Stammers et al., 2020). The present study findings also suggest that stress may be particularly tied to adolescents' use of eating for regulation of their negative emotions, a task they may be finding particularly difficult at this time of life (Skinner & Zimmer‐Gembeck, 2016).

The findings also support theory and research regarding the stress perceptions and problems that occur when eating is used in an attempt to manage dysregulation or to cope with stress (Michopoulos et al., 2015; Spoor et al., 2007; Stammers et al., 2020; Stice et al., 2002). The unexamined but implicit assumption is that EER reflects adolescents' attempts to seek comfort and cope after feeling stressed and threatened by daily problems. Such EER may be associated with other maladaptive emotion regulation strategies and ways of coping with stress. Consistent with this possibility, EER was positively related to avoidance and rumination in the present study (considered for EER measurement validation). Additionally, reliance on EER may displace other more effective and adaptive coping responses, such as talking to others, positive reappraisal, self‐compassion, or problem‐solving; all of which adolescents may struggle to put into practice. In sum, there are many reasons to suspect that EER will lead to even more feelings of being “stressed” over time.

Once adolescents' appraisals of stressor intensity and the psychological need threat from stressful events were considered, greater chronicity of stress was associated with a greater degree of EER on the same reporting day. However, chronicity was not uniquely associated with a greater degree of EER the following day. Some research has found chronicity to increase food consumption and emotional eating among adults (O'Connor et al., 2008; Stammers et al., 2020). However, studies rarely differentiate stressor intensity from chronicity; this is the first study that included a specific report of stress chronicity in a diary study alongside separate appraisals of stressor intensity and threat. This finding could be unique to adolescents, however. The specific problems reported by adolescents each day were not usually chronic—with stressful events, on average and most often, starting on the day it was reported. For adolescents, most of the stressful events they experienced seemed to be acute (they did not occur across days), rather than chronic. Given this, the findings should be interpreted as the role of acute daily stress in adolescents' EER.

### Stress by domain

Differentiating chronic and acute stress does raise an issue regarding how to capture important characteristics of a stressful event when conducting research, especially when using intensive repeated measures designs. A stressful event can be a particular event within a larger domain, such as a problem with homework in the academic domain, or it could be numerous problems within a domain over time, such as a problem with homework 1 day, a poor grade on a test the next day, etc. In this way, a single stressful event itself may not be chronic, but stress in a domain could be occurring repeatedly. In addition, some domains of stress may yield more or less intensity and threats to the psychological needs for relatedness, competence, and autonomy than other domains. Thus, the present study concentrated on stressor domain as one approach to addressing both chronicity and significance of stress in any one domain. Adolescents' ratings of intensity and threat within three domains of stress: peer relationships, family, and academics were compared. When intensity and threat within each stressor domain were correlated with EER, with one exception of peer stressor intensity, all stressor domains were linked to a greater degree of EER. However, there was also evidence that peer and family stressors (interpersonal stressors) may be more likely to yield elevated EER than academic stress (ego‐threatening stressors). When EER was compared on days with or without a stressor in each particular domain, EER was more elevated on days when peer or family stressful events were reported as adolescents' “biggest problem” compared to other days. This difference in EER was not found for academic stressful events.

The frequency of stress within each domain may be one explanation for these findings. Academic stress was the most commonly reported problem of the day for most adolescents. Thus, less common stressors, such as problems with peers or family, may be more likely to be linked to EER on the same day. The survey allowed for one problem to be reported, so peer and family stress could have been added on to the more common academic problems that occurred frequently for young people. Overall, however, the findings show that different stressor types (domains) all are related to EER in some way, as has been reported in some research with adults (Claus & Byrd‐Craven, 2019; see reviews by O'Connor et al., 2008; O'Connor & Conner, 2011).

### Limitations and future research

This study had the advantage of a 7‐day daily diary study collecting data on multiple appraisals of stressful events and information regarding the domains of stress, including a large group of adolescents. Nevertheless, there are some study limitations to mention. First, daily reports were collected across 7 days, and adolescents reported a single “biggest problem” towards the end of each day. This method has been used in multiple previous studies with success, but it also produced some disadvantages regarding understanding the timing of stress and eating. There is evidence that the end of the day may be a high‐risk time for cravings and consuming high‐fat foods (Haynes et al., 2016; Warde & Yates, 2017) and for stress‐related eating, even if the stress was much earlier in the day (Huh et al., 2015). Thus, collecting data in the evening is probably a good approach, but it still did not allow for analyses of the timing between stress and EER (or vice versa). For example, craving food may increase close to when a stressful event occurs, but the actual consumption of food may be delayed until it is available or when defenses break down (Chawner & Filippetti, 2024). Such availability may differ depending on the type of stressor and other circumstances. As described by Chawner and Filippetti (2024) in their developmental model of emotional eating, emotional eating is “not only a trait, but also a state‐like behavior that can change depending on the circumstances within which eating occurs” (p. 2). Such possibilities suggest that measuring stress and EER multiple times a day over more than a week could be useful for replicating and extending the current study findings.

A second limitation concerns stressor domain. Completing complex analyses within any specific stressor domain (e.g., peer stressors) was not possible in this study because of the variable frequency of stressors across days and adolescents. Thus, 7 days may not have been a long enough window of time to collect information on enough stressful events within every domain of interest to produce stable findings, making it important to replicate the within‐domain analyses reported here. In fact, this is the primary reason why dynamic structural equation modeling (DSEM) was not conducted for each stressor domain.

A third limitation in the present study was the lack of attention to moderators or important covariates. For example, research has identified multiple factors that can moderate associations of stress with eating or emotional eating. Studies report that stress is more strongly associated with eating behavior among those who are overweight or those who are restraining their eating compared to others (Cardi et al., 2015; Evers et al., 2018). Reichenberger et al. (2021) referred to this as an individual differences view of the stress‐eating association, whereby the association has long been expected (and often found) to differ among groups of individuals based on physiology, stress‐related social conditioning of eating behavior, or other differences. This may also be the case for associations of stress with EER. Similarly, body dissatisfaction and general mental health may be important covariates (or moderators) to consider in future research on stress and EER. Both body dissatisfaction and mental health are known correlates of disordered eating, and each may place adolescents at more risk of negative emotion and EER when stressful events occur. Measures of weight, eating or weight loss goals (e.g., restrained eating/dieting), and body dissatisfaction were not collected in this study. Future research could examine covariates or moderators. In addition, research could be conducted with selected groups who may report EER (or other aspects of emotional eating) or stress in their lives, especially given that stressor intensity and threat were rather low (on average) in the current study, whereas EER was moderate.

The final limitation relates to the use of new items in the daily survey. Although all items were closely matched to survey items used in past research, the specific items measuring stress appraisals and the single item to measure EER had not been used in a previous study or validated prior to their use in this study. EER was measured with a single item designed to gather information about daily eating alongside emotion regulation as a motivation for eating. In addition, the response options for the EER item were founded on previous measures of emotion regulation strategies and ways of coping with stress, but they could be better aligned with the wording of the EER item. More generally, there can be limitations to using single items relative to multiple‐item measures, but evidence shows that single items embedded in ecological momentary assessments perform well in many cases (Song et al., 2023). Despite this evidence, future research is needed to validate new items, especially the item used to measure EER, and to test alternative items or additional items in research on emotional eating or EER among adolescents.

## CONCLUSION

Findings from this daily diary of adolescents indicate that eating for the purpose of emotion regulation (*EER*) and stressor intensity and threat are intertwined: daily stress provokes a higher degree of EER the following day and, conversely, EER on 1 day increases perceived stressor intensity and threat the following day. Additionally, although academic stress was commonly reported by adolescents across the days, interpersonal stress—both stress in peer and family relationships—emerged as a particular risk context for EER. Considered in conjunction with prior research on emotion regulation and disordered eating, these findings suggest that stress, especially interpersonal stressors, may heighten adolescents' risk for later disordered eating and adjustment difficulties through reliance on eating to alleviate or soothe negative affect.

## AUTHOR CONTRIBUTIONS

**Melanie J. Zimmer‐Gembeck:** Conceptualization; methodology; data curation; formal analysis; funding acquisition; visualization; project administration; writing – original draft; writing – review and editing.

## FUNDING INFORMATION

This research was funded by the Australian Research Council Discovery Grant (DP190101170).

## CONFLICT OF INTEREST STATEMENT

The author has no conflicts of interest to disclose.

## ETHICAL APPROVAL

Approval for this study was received from the Griffith University Human Research Ethics Committee (Protocol 2019/178).

## CONSENT STATEMENT

Parents provided written informed consent for participation and for the publication of anonymized data in this manuscript.

## Data Availability

The data that support the findings of this study are available from the corresponding author upon reasonable request.
